# N-Methyl-D-aspartate Receptor Excessive Activation Inhibited Fetal Rat Lung Development* In Vivo* and* In Vitro*

**DOI:** 10.1155/2016/5843981

**Published:** 2016-07-10

**Authors:** Zhengchang Liao, Xiaocheng Zhou, Ziqiang Luo, Huiyi Huo, Mingjie Wang, Xiaohe Yu, Chuanding Cao, Ying Ding, Zeng Xiong, Shaojie Yue

**Affiliations:** ^1^Department of Pediatrics, Xiangya Hospital, Central South University, Changsha, Hunan 410008, China; ^2^Department of Pediatrics, The First Hospital of Hunan University of Chinese Medicine, Changsha, Hunan, China; ^3^Department of Physiology, Xiangya Medical School, Central South University, Changsha, Hunan 410008, China; ^4^Department of Radiology, Xiangya Hospital, Central South University, Changsha, Hunan 410008, China

## Abstract

*Background*. Intrauterine hypoxia is a common cause of fetal growth and lung development restriction. Although N-methyl-D-aspartate receptors (NMDARs) are distributed in the postnatal lung and play a role in lung injury, little is known about NMDAR's expression and role in fetal lung development.* Methods*. Real-time PCR and western blotting analysis were performed to detect NMDARs between embryonic days (E) 15.5 and E21.5 in fetal rat lungs. NMDAR antagonist MK-801's influence on intrauterine hypoxia-induced retardation of fetal lung development was tested* in vivo*, and NMDA's direct effect on fetal lung development was observed using fetal lung organ culture* in vitro*.* Results*. All seven NMDARs are expressed in fetal rat lungs. Intrauterine hypoxia upregulated NMDARs expression in fetal lungs and decreased fetal body weight, lung weight, lung-weight-to-body-weight ratio, and radial alveolar count, whereas MK-801 alleviated this damage* in vivo*.* In vitro *experiments showed that NMDA decreased saccular circumference and area per unit and downregulated thyroid transcription factor-1 and surfactant protein-C mRNA expression.* Conclusions*. The excessive activation of NMDARs contributed to hypoxia-induced fetal lung development retardation and appropriate blockade of NMDAR might be a novel therapeutic strategy for minimizing the negative outcomes of prenatal hypoxia on lung development.

## 1. Introduction

Prenatal hypoxia is a common clinical phenomenon and cause of fetal growth and lung development restriction [1]. Lung development, which starts during the fetal period and continues postnatally, is highly susceptible to hypoxia-induced damage.

The rat lungs undergo several stage-specific changes during gestation. Histologically, these stages include embryonic stage (E9–E11.5, embryonic day (E)), pseudoglandular stage (E11.5–E18.5), canalicular stage (E18.5–E19.5), saccular stage (E19.5–PN7, postnatal day (PN)), and alveolar stage (PN7–35). The histomorphological processes occurring during lung development include branching, cellular differentiation and proliferation, and alveolarization [2, 3]. Accumulating evidence suggests that exposure to hypoxia during the prenatal period affects the structure and function of the respiratory system. For instance, in pregnant rats kept in a hypoxic atmosphere of 13% O_2_ throughout gestation, septation of saccules is attenuated and alveoli number is reduced in rat pups [4]. Perinatal hypoxia also disrupts pulmonary vascular growth and increases hypoxic pulmonary vasoconstriction in offspring [5].

N-Methyl-D-aspartate receptors (NMDARs) are ionotropic glutamate receptors. The NMDAR family is composed of seven subunits: NMDAR1, NMDAR2A, NMDAR2B, NMDAR2C, NMDAR2D, NMDAR3A, and NMDAR3B [6]. NMDARs have been characterized by their roles in the central nervous system and their involvement in neuronal excitotoxicity in neurological disorders [7]. Recently, it has become clear that NMDARs are also functional in nonneuronal tissues and are widely distributed in the pancreas, skin, bone, gastrointestinal tract, liver, heart, and kidney [8–10]. It has also been reported that NMDARs are distributed in the adult lung [11].

Our previous studies confirmed that the NMDAR2D existed in the newborn rat lungs [12]. Hyperoxia and bleomycin could enhance lung endogenous glutamate release, and NMDAR antagonist attenuated lung injury induced by hyperoxia and bleomycin, respectively [12–14]. Said et al. also report that NMDA induces excitotoxicity in perfused rat lungs in the form of acute, high-permeability pulmonary edema [15]. These studies indicate that NMDARs play important roles in lung injury.

However, it is currently still unclear whether NMDARs exist in the fetal lungs and whether their excessive activation disrupts fetal lung development. Our aim of this study was to determine the role of NMDARs overactivation in prenatal hypoxia-induced retardation of fetal lung development. First, real-time PCR and western blotting analysis were performed to detect all NMDAR subunits expressions in the rat lungs on E15.5 to E21.5. Next, the changes of NMDAR expression in the fetal lung on E21.5 and the effects of NMDAR antagonist MK-801 on fetal lung development were investigated after intrauterine hypoxia exposure. Finally, through an* in vitro *E15.5 whole fetal lung organ culture model, the direct effects of NMDA on fetal lung development were investigated. NMDA's effects on thyroid transcription factor-1 (TTF-1, a key transcription regulator of pulmonary morphogenesis [16]) and surfactant protein-C (SP-C, a type II alveolar epithelial cell marker) mRNA expressions were detected to study their possible underlying mechanism.

## 2. Materials and Methods

### 2.1. Animals

Female Sprague-Dawley rats (200–250 g) and male Sprague-Dawley rats (300–350 g) were purchased from the Animal Center of Central South University, Changsha, China. Rats were acclimated for 4-5 days and mated within the animal facility. The day on which vaginal plugs were observed was considered E0.5.

Pregnant rats were randomly assigned to one of four groups on E15.5: (1) rats in the air control group were maintained in normoxic conditions; (2) rats in the air + MK-801 group were maintained in normoxic conditions and received daily intraperitoneal (i.p.) injections of 0.05 mg/kg MK-801 (Sigma, St. Louis, MO); (3) rats in the hypoxia group were exposed to hypoxic conditions (FiO_2_ = 0.105) for 8 hr/day from E15.5 to E20.5; and (4) rats in the hypoxia + MK-801 group received daily i.p. injections of 0.05 mg/kg MK-801 before hypoxia exposure. Rats in the air control and hypoxia groups received the same volume of saline. On E21.5, pregnant rats were anesthetized with chloral hydrate, embryos and fetuses were removed, and fetal lungs were dissected. Every effort was made to minimize animal suffering. All animal procedures were reviewed and approved by the Committee on Research Animal Welfare of Central South University, Changsha, China.

### 2.2. *In Vitro *Whole Fetal Lung Organ Culture

Embryonic lungs were cultured as previously described [17]. Lung buds were dissected from E15.5 rat embryos and placed on translucent polycarbonate culture dish inserts (3.0 *μ*m pore size, 12 mm diameter; Transwell, Costar, UK) and cultured with 1000 *μ*L serum-free BGJb (Gibco, Grand Island, NY) supplemented with 100 U/mL penicillin and 100 *μ*g/mL streptomycin. Lung cultures were randomly divided into control group and NMDA-treated group. NMDA (M3262, Sigma, St. Louis, MO, USA) was added to the medium at a final concentration of 1 mmol/L. Both groups were incubated at 37°C in an atmosphere containing 21% O_2_ and 5% CO_2_. Organ cultures were maintained for 96 hr. The medium was changed every other day.

### 2.3. Morphometric Analysis

E21.5 fetal lungs and E15.5 fetal lungs were cultured for 48 or 96 hr and then harvested, fixed in 4% paraformaldehyde, and processed into serial paraffin sections using routine procedures. Sections were stained with hematoxylin and eosin, examined under a light microscope, and photographed. Morphometric analyses were conducted using ImageJ software (National Institutes of Health, Bethesda, MD). Saccular circumference per unit and saccular area per unit were determined. Radial alveolar counts (RAC) were also determined as previously described [18]. All analyses were performed by an independent pathologist who was blind to group identity.

### 2.4. Total RNA Extraction, Reverse Transcription, and Real-Time PCR

E15.5–E21.5 fetal lungs were cultured for 24, 48, 72, or 96 hr, harvested, and immediately frozen in liquid nitrogen until use. Total RNA was isolated from frozen lung samples with TRIzol reagent (Sigma) based on the manufacturer's instructions. Reverse transcription was performed with 2 *μ*g total RNA using a kit from Promega (Madison, WI) following the manufacturer's instructions. Real-time PCR was performed using iTaq SYBR Green Supermix with Rox (Bio-Rad, Hercules, CA).

Reactions were performed in triplicate according to the manufacturer's instructions with 200 nM of each sense and antisense primer for a final reaction volume of 25 *μ*L/well.

Primers used in real-time PCR experiments are shown in Table 1.

### 2.5. Western Blotting Analysis

The fetal lung tissues were collected and stored at −80°C until use. Western blotting analyses of fetal lung tissues were performed as previously described [19]. The primary antibodies used were as follows: NMDAR1 (rabbit polyclonal antibody, 1 : 1000) (Cell Signaling Technology, USA), NMDAR2D (rabbit polyclonal antibody, 1 : 200) (Bioss, China), NMDAR3A (rabbit polyclonal antibody, 1 : 200) (Bioss, China), and *β*-actin (mouse polyclonal antibody, 1 : 4000) (Proteintech, USA). Finally, these were followed by incubation with the horseradish peroxidase- (HRP-) conjugated secondary antibodies (dilution: 1 : 3000) (Proteintech, USA) for 1 hr. *β*-actin was used as an internal control for the semiquantitative assay.

### 2.6. Statistical Analysis

All data are shown as mean ± standard deviation. Student's *t*-tests and one-way analysis of variance (ANOVA) were performed using SPSS 15.0 statistical software (SPSS Inc., Chicago, IL). Statistical significance was set at *p* < 0.05.

## 3. Results

### 3.1. Prenatal Hypoxia Increased NMDAR Subunits mRNA Levels and Protein Expressions in Fetal Lungs

**Figure 1:**
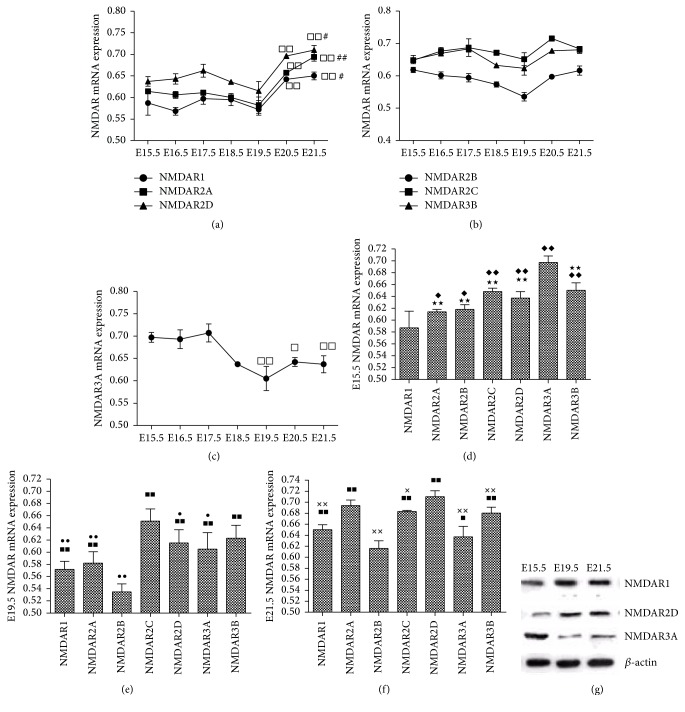
(a)–(f) NMDARs mRNA expression in fetal rat lung. Fetal rat lungs expressed mRNA for all seven NMDAR subunits between E15.5 and E21.5. (a) NMDAR1, NMDAR2A, and NMDAR2D mRNA expression in fetal rat lung development. (b) NMDAR2B, NMDAR2C, and NMDAR3B mRNA expression in fetal rat lung development. (c) NMDAR3A mRNA expression in fetal rat lung development. (d) All the seven NMDARs' mRNA expression on E15.5. (e) All the seven NMDARs' mRNA expression on E19.5. (f) All the seven NMDARs' mRNA expression on E21.5.  ^□^ *p* < 0.05 and ^□□^ *p* < 0.01 versus E15.5; ^#^ *p* < 0.05 and ^##^ *p* < 0.01 versus E15.5; ^*◆*^ *p* < 0.05 and ^*◆◆*^ *p* < 0.01 versus NMDAR1; ^★★^ *p* < 0.01 versus NMDAR3A; ^■^ *p* < 0.05 and ^■■^ *p* < 0.01 versus NMDAR2B; ^●^ *p* < 0.05 and ^●●^ *p* < 0.01 versus NMDAR2C; ^×^ *p* < 0.05 and ^××^ *p* < 0.01 versus NMDAR2D. (g) NMDARs protein expression in fetal rat lungs. E: embryonic day.

NMDARs' mRNAs were measured in fetal rat lungs between E15.5 and E21.5. Based on the expression similarities, the seven NMDAR subunits' expression during fetal lung development could be categorized with three different patterns. Pattern 1 included NMDAR1, NMDAR2A, and NMDAR2D—whose mRNA levels increased across lung development, with significantly higher mRNA levels on E21.5 than on E15.5 (Figure 1(a)). Pattern 2 included NMDAR2B, NMDAR2C, and NMDAR3B—whose mRNA levels remained relatively steady across lung development except on E19.5, when mRNA levels were downregulated (Figure 1(b)). Pattern 3 included NMDAR3A—whose mRNA level decreased across development (Figure 1(c)). On E15.5, NMDAR3A mRNA level was generally higher than that of other subunits, and NMDAR1 mRNA level was lower than that of other subunits (Figure 1(d)). On E19.5, NMDAR2C mRNA level was generally higher than those of other subunits (Figure 1(e)). On E21.5, NMDAR2A and NMDAR2D were predominant subunits (Figure 1(f)).

We further determined the changes in protein levels of NMDARs during fetal lung development. We chose NMDAR1, NMDAR2D, and NMDAR3A protein expression on E15.5, E19.5, and E21.5 as representatives. As shown in Figure 1(g), the protein levels of NMDAR1 and NMDAR2D were low at E15.5 and gradually increased and finally reached peak at E21.5. The protein level of NMDAR3A was high at E15.5, followed by a gradual decline in expression. The patterns of protein expression were similar to their respective mRNA expression pattern.

**Figure 2:**
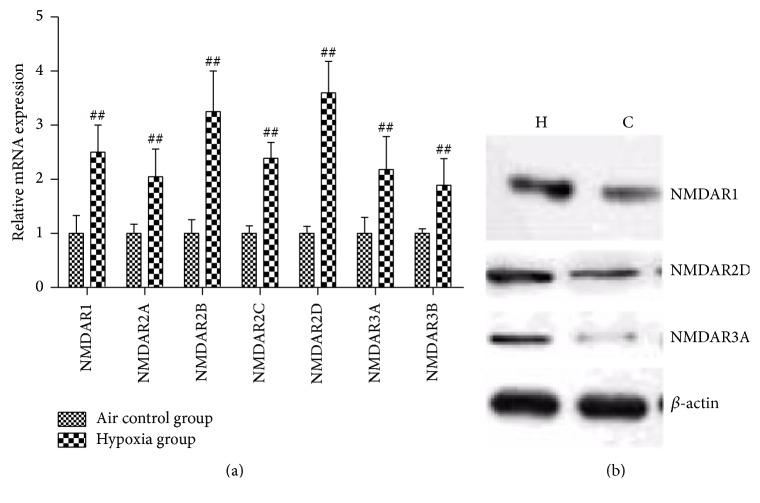
(a) NMDAR mRNA expression in fetal rat lungs after 6 days of intrauterine hypoxia. Intrauterine hypoxia enhanced NMDAR mRNA expression in fetal lungs. ^##^ *p* < 0.01 versus air control group. (b) NMDAR protein expression in fetal rat lungs after 6 days of intrauterine hypoxia. H: hypoxia group; C: control group.

We next exposed pregnant rats to hypoxia (FIO_2_ = 0.105, 8 hr/day) from E15.5 to E20.5. Interestingly, on E21.5, NMDARs mRNA levels in fetal rat lungs from the hypoxia group were found to be significantly higher than those from the air control group (*p* < 0.01; Figure 2(a)). It was also observed that NMDAR1, NMDAR2D, and NMDAR3A protein expressions increased after hypoxia exposure (Figure 2(b)). These results indicated that intrauterine hypoxia enhanced fetal lung NMDARs transcriptions and translations.

### 3.2. MK-801 Prevented Hypoxia-Induced Impairment of Fetal Lung Development* In Vivo*

**Figure 3:**
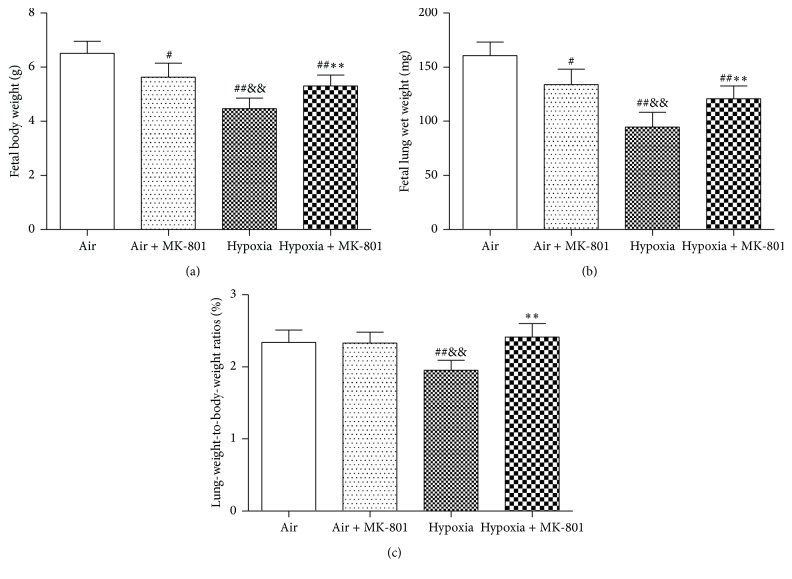
Effect of MK-801 on (a) fetal rat body weight, (b) fetal lung wet weight, and (c) lung-weight-to-body-weight ratio after 6 days of intrauterine hypoxia. Intrauterine hypoxia induced the decrease in body weight, lung wet weight, and lung-weight-to-body-weight ratio; MK-801 administration before hypoxia alleviated the decrease in body weight, lung wet weight, and lung-weight-to-body-weight ratio in fetal hypoxic rats. ^#^ *p* < 0.05 and ^##^ *p* < 0.01 versus air control group; ^&&^ *p* < 0.01 versus air + MK-801 group; ^*∗∗*^ *p* < 0.01 versus hypoxia group.

To investigate the significance of higher expressions of fetal lung NMDARs after intrauterine hypoxia exposure, the effects of NMDAR antagonist MK-801 on fetal body and lung weight in intrauterine hypoxia status were examined. On E21.5, body weight, lung wet weight, and lung-weight-to-body-weight ratio were significantly lower in fetal rats in the hypoxia group than in the air control group (*p* < 0.01) (Figure 3).

**Figure 4:**
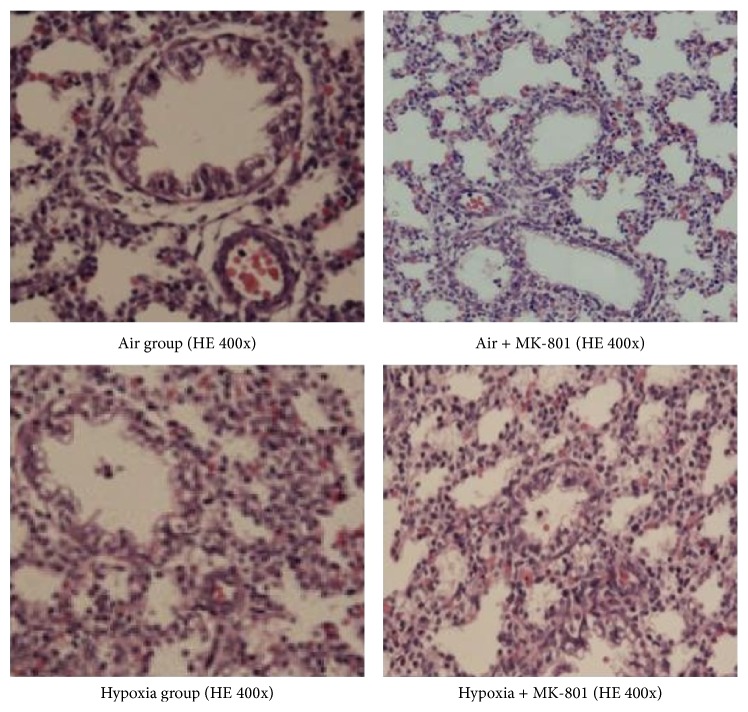
Effect of MK-801 on histological structures of E21.5 fetal rat lungs after 6 days of intrauterine hypoxia exposure (hematoxylin and eosin staining, 400x magnification).

**Figure 5:**
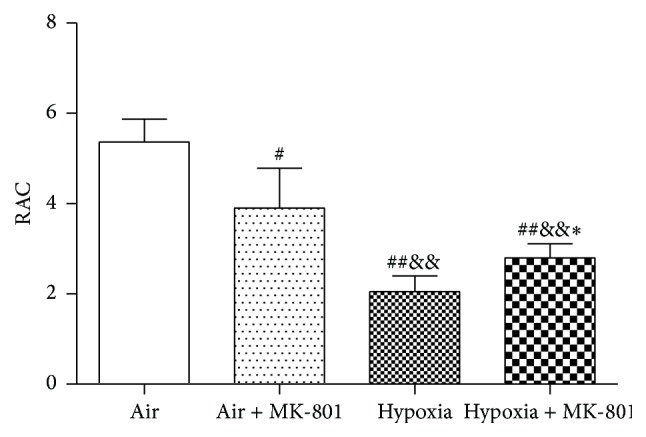
Effect of MK-801 on RAC in fetal rat lungs after 6 days of intrauterine hypoxia exposure. Intrauterine hypoxia induced the decrease in RAC; MK-801 administration alleviated the hypoxia-induced RAC decrease. ^#^ *p* < 0.05 and ^##^ *p* < 0.01 versus air control group; ^&&^ *p* < 0.01 versus air + MK-801 group; ^*∗*^ *p* < 0.05 versus hypoxia group.

MK-801 alleviated the decreases in body weight, lung wet weight, and lung-weight-to-body-weight ratio induced by intrauterine hypoxia (*p* < 0.01). However, body and lung wet weights of fetal rats in the hypoxia + MK-801 group were still lower than those in the air control group (*p* < 0.01), and there was no significant difference in lung-weight-to-body-weight ratio between fetal rats in the hypoxia + MK-801 group and the air control group. Histological observation revealed that fetal lungs in the hypoxia group appeared to have more irregularly shaped, fewer alveolar-like structures (Figure 4) and lower radial alveolar count (RAC) (*p* < 0.01; Figure 5) compared with the air control group. MK-801 alleviated hypoxia-induced lung histological change, showed more regularly shaped and more alveolar-like structures (Figure 4), and significantly increased RAC (*p* < 0.05; Figure 5). However, fetal rat body weight, lung weight, and RAC in air + MK-801 group were lower than in the air control group (*p* < 0.05; Figure 5). Taken together, it is suggested that overexpression of NMDAR correlates with abnormal fetal lung development and the blockage of overexpressed NMDARs could rescue lung development restrictions induced by hypoxia.

### 3.3. NMDA Inhibited Fetal Lung Development* In Vitro*

**Figure 6:**
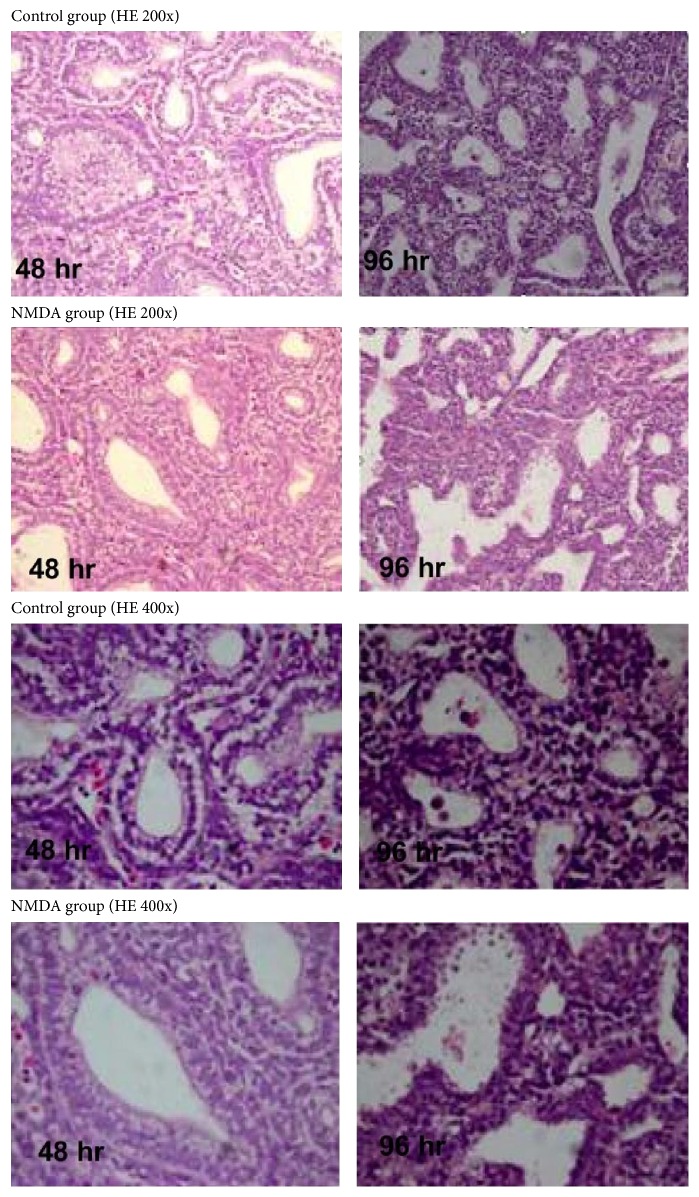
Effect of NMDA on histological structures of cultured fetal rat lungs (hematoxylin and eosin staining, upper rows: 200x magnification, lower rows: 400x magnification). In air group, airway tubes were lined with a highly columnar epithelium at 48 hr, and epithelial cells differentiated into a cuboidal epithelium at 96 hr. After administration of NMDA, the differentiation of epithelial cells from a highly columnar epithelium to a cuboidal epithelium was not visible. Also, with longer culture durations, the histological structures of NMDA-treated explants became irregular.

**Figure 7:**
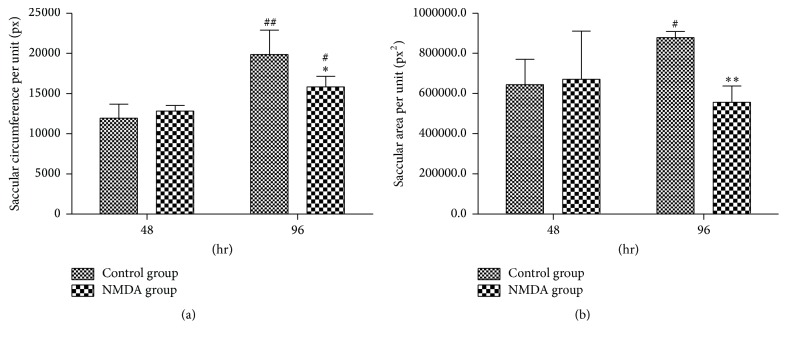
Effect of NMDA on (a) saccular circumference per unit and (b) saccular area per unit. NMDA decreased the saccular circumference per unit and saccular area per unit of lung explants at 96 hr. ^*∗*^ *p* < 0.05 and ^*∗∗*^ *p* < 0.01 versus control group at the same time point; ^#^ *p* < 0.05 and ^##^ *p* < 0.01 versus the same group at 48 hr.

To demonstrate the direct effect of excessive NMDAR activation on fetal lung development, NMDA's effect on* in vitro *cultured fetal lung was examined. E15.5 lung primordia were cultured for up to 96 hr in the absence of serum or humoral factors. In the control group, saccular circumference per unit (Figure 7(a)) and saccular area per unit (Figure 7(b)) were significantly increased at 96 hr compared to at 48 hr (*p* < 0.05). Histological observation of control lung explants showed that airway tubes were lined with a highly columnar epithelium at 48 hr and epithelial cells differentiated into a cuboidal epithelium at 96 hr (Figure 6). This indicated that fetal lungs developed gradually* in vitro *with the elongation of culture time. The results were similar to previously reported studies [17]. Following administration of NMDA (1 mmol/L), the saccular circumference per unit and area per unit were significantly reduced at 96 hr compared with control group (*p* < 0.05) (Figure 7). And histological observation of lung explants in NMDA group showed more irregularly shaped structures. The results suggest that NMDA treatment inhibited fetal rat lung development* in vitro*.

### 3.4. NMDA Downregulated Surfactant Protein-C (SP-C) and Thyroid Transcription Factor-1 (TTF-1) mRNA Expressions in Fetal Lung Explants* In Vitro*

**Figure 8:**
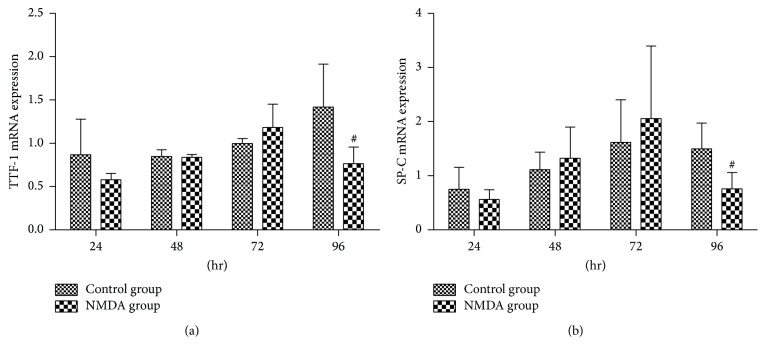
Effect of NMDA on (a) TTF-1 mRNA expression and (b) SP-C mRNA expression. NMDA decreased TTF-1 and SP-C mRNA expression at 96 hours. ^#^ *p* < 0.05 versus control group at the same time point.

Type II alveolar epithelial cell (AEC II) not only secretes pulmonary surfactant, but also transdifferentiates into type I alveolar epithelial cell (AEC I), which is critical for reestablishment and maintenance of an intact alveolar epithelium [20]. Failure of functional maturation of AEC II during alveologenesis can induce a dramatic reduction in surfactant protein resulting in neonatal respiratory distress [21]. Surfactant protein-C (SP-C) is often used as a marker of alveolar type II epithelial cell maturation and is expressed early in fetal lung development (at E12.5). SP-C expression is initially restricted to the termini of epithelial tubules and increases with the growth of the fetus [22]. TTF-1 (also termed Nkx2.1 or T-EBP) is one of the most important transcription factors which could regulate alveolar epithelial cell development [23]. In this study, SP-C and TTF-1 mRNA expressions were detected in E15.5 fetal lungs cultured for 24, 48, 72, and 96 hr. At 96 hr, SP-C and TTF-1 mRNA expressions were significantly downregulated in the NMDA-treated group (*p* < 0.05) (Figure 8). These findings suggested that overactivation of NMDAR could inhibit fetal lung type II alveolar epithelial cells development.

## 4. Discussion

It has been well reported that NMDAR subunits are expressed in the fetal brain and mediate a variety of developmental functions [6, 24]. In this study, we found that fetal rat lungs also expressed NMDARs, intrauterine hypoxia exposure enhanced the expression of NMDARs, and MK-801 alleviated intrauterine hypoxia-induced fetal lung growth retardation, including the impaired fetal alveoli formation* in vivo*. NMDA also inhibited the development of fetal lung explants* in vitro*.

NMDA receptors are heteromeric complexes comprising an NR1 subunit combined with one or more NR2 or NR3 subunits. The NMDA receptor system has been shown to play a major role in the normal development of the CNS. This development occurs through many stages of neurogenesis, migration, proliferation, neuronal apoptosis, axonal outgrowth, and synapse formation and elimination [25]. It was reported that NMDARl mRNA, barely detectable at embryonic day 14 in the brain, increased gradually during development until the third postnatal week and then it declined slightly to adult levels in rat brain [26]. NMDAR2A, NMDAR2B, and NMDAR2C were observed at E18. NMDAR2A and NMDAR2C increased gradually during brain development, while NMDAR2B reached the peak expression at p4 (p: postnatal day) and was hardly observed at p7 and p14 in the rat cortex [27]. The NMDA receptors are ionotropic receptors, which have a cation-selective ion channel that regulates Na^+^, K^+^, and Ca^2+^. In the brain development, NMDAR activation is a double-edged sword. Physiological activation of NMDARs could promote survival of neuronal populations, such as cerebellar granule cells [28]; NMDAR overactivation could also lead to Na^+^ and Ca^2+^ influx resulting in neuronal apoptosis or necrosis [29, 30]. In summary, too much or too little NMDAR activation could result in neuronal cell death and could even be life-threatening during brain development [31]. But little is known about NMDAR's role in fetal lung development.

Some studies found that homozygous null mutant mice (NMDAR1^−/−^) died 8–15 hr after birth. Subsequent pathological analysis revealed that respiratory failure was the ultimate cause of death [32], suggesting that NMDARs were essential for embryonic respiratory development. In our research, we first showed that fetal rat lung expressed seven NMDAR subunits even though the NMDAR subunits expression patterns during fetal lung development were not exactly the same as that in fetal brain development. From E15.5 to E21.5, expression of NMDAR2B, NMDAR2C, and NMDAR3B remained steady while expression of NMDAR1, NMDAR2A, and NMDAR2D mRNA increased with fetal lung development.

A previous study reported that NMDAR2D was the dominant subtype expressed in the adult lung [11]. We also showed that NMDAR2D expression tended to increase across gestation and peaked at E21.5 before birth. It was also discovered that, after 6 days of hypoxia exposure, NMDARs mRNA and protein were significantly upregulated in hypoxic fetal rat lungs. Apparently, the physiological significance and pathophysiological significance of NMDARs expression level change in fetal lung development and fetal lung NMDARs upregulation in intrauterine hypoxia need to be investigated.

It has been widely accepted that hypoxia during pregnancy induces intrauterine growth restriction (IUGR) and can cause maldevelopment of the kidney, pancreas, cardiopulmonary system, and brain [33–36]. Our results showed that prenatal hypoxia reduced fetal lung wet weight and impaired fetal lung saccular formation in addition to inducing IUGR. Furthermore, lung-weight-to-body-weight ratio was also decreased in the hypoxia group, indicating that hypoxia exposure inhibited lung development more than body growth.

In order to explore the significance of NMDARs high expression in fetal lung development restriction induced by intrauterine hypoxia, the effects of MK-801, a NMDAR antagonist, were tested in the pregnant rat. The results showed that blocking NMDARs with MK-801 alleviated this hypoxia-induced retardation of lung development and improved the body weight, lung wet weight, lung-weight-to-body-weight ratio, and RAC, first demonstrating that excessive activation of NMDARs participated in hypoxia-induced fetal lung development retardation. Fetal rats in the air + MK-801 group also showed a significant reduction in body weight, lung wet weight, and RAC compared with fetal rats in the control group. This may suggest that NMDARs' normal expression and normal activation play an important role in fetal lung development, similar to NMDARs' normal physiologic role in fetal brain development. Too much or too little NMDAR activity could be harmful for fetal lung development. However, the mechanism of NMDAR excessive activation in fetal lung development retardation remained unclear.

Early-stage fetal lungs can develop in serumless, chemically defined medium with normal spatial and temporal patterns* in vitro* [17, 22], and the cultured embryonic lung is widely used to identify regulators of lung development. In previous* in vivo* studies, fetal lung saccular circumference and area per unit increased as pregnancy progressed [37], and the fetal rat lung at E15.5 is in a pseudoglandular period, with epithelial cells progressively differentiating from a columnar epithelium to a cuboidal epithelium [2]. We observed that fetal lung development under control conditions* in vitro* was similar to that* in vivo*. NMDA is one of the amino acid derivatives, and it is an important excitatory neurotransmitter L-glutamate analogue in the mammalian central nervous system. In organotypic hippocampal slice cultures* in vitro*, 0.15 mmol/L NMDA application rapidly induced excitotoxic cell damage and death [38]. Said et al. reported that 1 mmol/L NMDA induced acute high-permeability pulmonary edema* in vitro *[15]. To study the role of NDMARs' excessive activation in fetal lung development, we observed the effects of NMDA (1 mmol/L) on fetal lung development in* in vitro* organ culture model. After administration of NMDA, saccular circumference and area per unit were lower than those in the control group after 96 hr, and fetal lung histological structures were more irregularly shaped, indicating that NMDA inhibited fetal lung development* in vitro*.

Temporal and spatial control of gene expression of transcriptional factors is a hallmark of lung development. The program of lung development from its inception is directed by the activity of key transcriptional factors [39]. TTF-1 is a homeodomain transcriptional regulator that is expressed in the lung, the thyroid, and the ventral forebrain. In the lung, TTF-1 is essential for the expression of SP-A (surfactant protein-A), SP-B, and SP-C, CCSP (Clara cell secretory protein), Bmp4, *α*-integrins, and collagen type IV [40]. TTF-1 activity plays an important role in the distal lung morphogenesis. Morphologically, Nkx2.1^−/−^ (also known as TTF-1) lung epithelium is composed solely of stratified and pseudostratified columnar epithelial cells, which are characteristically expressed in proximal compartments of wild-type lungs.

Furthermore, distal lung epithelial-specific differentiation markers (e.g., SP-C and SP-A) are absent. All of these data suggest that Nkx2.1^−/−^ lungs are arrested in a proximal state [39]. Additionally, it has been reported that mice lacking TTF-1 protein die at birth because of respiratory distress [41]. And,* in vivo*, it has been demonstrated that prenatal hypoxia inhibited SP-C and TTF-1 mRNA expression [5, 42]. In this study, our results showed that TTF-1 and SP-C mRNAs were expressed in the fetal rat lung* in vitro* with similar temporal patterns to those observed* in vivo *[22, 43]. NMDA treatment for 96 hr decreased TTF-1 and SP-C expression and inhibited fetal lung development, implying that NMDA may impair fetal lung development through the downregulation of TTF-1 and SP-C expression.

We believe that NMDARs are closely involved in the lung development; we also understand that all the correlations discussed above still could not shed light on the direct mechanisms underlying the fetal lung development retardation associated with the NMDAR excessive activation. We did notice that Truog et al. studied the postnatal lung development in the chronic hypoxia status using DNA microarray and observed altered gene profiling correlated with the lung development and remodeling [18]. Clearly, expression changes of NMDARs were not included in this report.

Nevertheless, we are currently focusing on the high throughput studies using DNA microarray to investigate the gene expression profiling during the lung development in the prenatal stages in both normoxia and hypoxia conditions, attempting to discover critical genes as well as pathways that are responsible for the hampered fetal lung development in hypoxia status.

## 5. Conclusion

In conclusion, our research is the first report showing that NMDARs are expressed in the fetal rat lungs from E15.5 and E21.5, NMDARs' expressions are upregulated by intrauterine hypoxia exposure, and excessive activation of NMDARs could impair lung development* in vivo *and* in vitro*. All of these suggest that excessive activation of NMDARs is involved in fetal lung development restriction induced by intrauterine hypoxia. Since NMDARs' normal expression and activation are very important in normal fetal brain and lung development, NMDAR antagonist's application in prevention and cure of fetal lung development restriction induced by intrauterine hypoxia needs to be further addressed.

## Figures and Tables

**Table 1:** Primer sequences for real-time PCR.

Genes	Forward	Reverse
*Nmdar1*	CAGGAGTGGAACGGAATCAT	ACTTGAAGGGCTTGGAGAAC
*Nmdar2a*	AGCCATTGCTGTCTTCGTTT	ATCTTGCTGGTTGTGCCTTT
*Nmdar2b*	GCGATAATGGCGGATAAGGA	AGGTAGGTGGTGACGATGGAA
*Nmdar2c*	CACACCCACATGGTCAAGTTC	ATGGTGACCAGCTTGCAGC
*Nmdar2d*	CGAGGATGGCTTTCTGGTGA	ATACTTGAGGCGGAGGGTCTG
*Nmdar3a*	GCGGGATGCCCTACTGTT	CATTTCGCCCTGGCTCTG
*Nmdar3b*	CGAGGATGGCTTTCTGGTGA	ATACTTGAGGCGGAGGGTCTG
*Ttf-1*	CTCACCGCTTTCCCTCTACC	ATACTTGAGGCGGAGGGTCT
*Spc*	CTTGTCGTCGTGGTGATTGTA	AAGGTAGCGATGGTGTCTGTG
*β-actin*	TGACGTGGACATCCGCAAAG	CTGGAAGGTGGACAGCGAGG
